# Covid-19 and Growth Hormone/Insulin-Like Growth Factor 1: Study in Critically and Non-Critically Ill Patients

**DOI:** 10.3389/fendo.2021.644055

**Published:** 2021-06-16

**Authors:** Ioannis Ilias, Aristidis Diamantopoulos, Efthymia Botoula, Nikolaos Athanasiou, Alexandros Zacharis, Stamatios Tsipilis, Edison Jahaj, Alice G. Vassiliou, Dimitra A. Vassiliadi, Anastasia Kotanidou, Stylianos Tsagarakis, Ioanna Dimopoulou

**Affiliations:** ^1^ Department of Endocrinology, Diabetes and Metabolism, Elena Venizelou Hospital, Athens, Greece; ^2^ Department of Endocrinology, Diabetes and Metabolism, Evagelismos Hospital, Athens, Greece; ^3^ First Department of Critical Care Medicine & Pulmonary Services, Medical School of National & Kapodistrian University of Athens, Evagelismos Hospital, Athens, Greece; ^4^ Department of Pulmonary Medicine, Evagelismos Hospital, Athens, Greece

**Keywords:** growth hormone, insulin-like growth factor 1, pandemics, severe acute respiratory syndrome coronavirus 2, humans, hospitalization

## Abstract

**Objective:**

We aimed to measure insulin-like growth factor 1 (IGF1) and growth hormone (GH) in critically and non-critically ill patients with Covid-19 and assess them vis-a-vis clinical and laboratory parameters and prognostic tools.

**Subjects and Methods:**

We included patients who were admitted to the wards or the ICU of the largest Covid-19 referral hospital in Greece; patients with non-Covid-19 pneumonia served as controls. Apart from the routine laboratory work-up for Covid-19 we measured GH and IGF1 (and calculated normalized IGF-1 values as standard deviation scores; SDS), after blood sampling upon admission to the wards or the ICU.

**Results:**

We studied 209 critically and non-critically ill patients with Covid-19 and 39 control patients. Patients with Covid-19 who were ICU non-survivors were older and presented with a worse hematological/biochemical profile (including white blood cell count, troponin, glucose, aminotransferases and lactate dehydrogenase) compared to ICU survivors or Covid-19 survivors in the wards. Overall, IGF-1 SDS was higher in Covid-19 survivors compared to non-survivors (-0.96 ± 1.89 vs -2.05 ± 2.48, respectively, p=0.030). No significant differences were noted in GH between the groups. Nevertheless, in critically ill patients with Covid-19, the prognostic value of IGF-1 (raw data), IGF-1 (SDS) and GH for survival/non-survival was on a par with that of APACHE II and SOFA (with a marginal difference between GH and SOFA).

**Conclusion:**

In conclusion, our findings suggest that there might be an association between low IGF1 (and possibly GH) and poor outcome in patients with Covid-19.

## Introduction

The novel coronavirus, severe acute respiratory syndrome coronavirus 2 (SARS-CoV-2), affects primarily the respiratory tract, but may also cause multi-organ dysfunction due to the widespread presence of angiotensin-converting enzyme-2 (ACE-2) receptors, an entry point for the virus (1). As far as the endocrine glands are concerned, changes in thyroid, pancreas, adrenals and gonads are increasingly being reported (2). Despite these, no clinical study has investigated the potential link between SARS-CoV-2 and the growth hormone (GH)/insulin-like growth factor-1 (IGF1) axis. A study has been done on laboratory results of Covid-19 patients from the UK Biobank (3) and another molecular study has been done with few patients (n=20) (4). Recently, speculation on diminished GH action in patients with Covid-19 has been put forth in this Journal (5). In this observational study we measured IGF1/GH in patients with Covid-19, hospitalized in the ward or the Intensive Care Unit (ICU) and assessed their levels vis-a-vis clinical and laboratory parameters and prognostic tools.

## Subjects and Methods

In this study we included only patients with Covid-19 (verified with polymerase chain reaction; PCR) who were admitted - from April 2020 to October 2020 - to the wards or the ICU of the largest Covid-19 referral hospital (with approximately 1000 hospital beds in total) in Athens, Greece. Patients in other wards with non-Covid-19 pneumonia served as contemporaneous controls. Patients receiving dexamethasone were excluded. Apart from the routine laboratory work-up for Covid-19 we also measured morning GH and IGF1 within 48 hours of admission to the wards or the ICU (GH and IGF1 were measured with the Immulite 2000 hGH and IGF1 Assays and Analyzer, Siemens Healthcare GmbH, 91052 Erlangen, Germany). We also normalized IGF-1 values by age/gender and expressed them as standard deviation scores (SDS), per the procedure described by Chanson et al. (6) (http://ticemed_sa.upmc.fr/sd_score/gestion_site/index_en.php). This was an observational study and the noted endpoint was uniquely hospital survival/mortality. Comparisons of parameters among four or six study groups (Covid-19 ICU survivors & non-survivors, Covid-19 wards survivors & non-survivors, Non-Covid-19 wards survivors & non-survivors) was done with analysis of variance (ANOVA), the Kruskall-Wallis test (according to the normality/non-normality of their distribution per the Kolmogorov-Smirnov test), whereas the Chi-Square test was used for categorical variables; Pearson’s test was used for correlations between parameters; statistical significance was set at p=0.05. A Receiver Operating Characteristic (ROC) curve plot for IGF1/IGF1 SDS and GH versus survival in ICU patients was constructed and Youden’s J index was calculated; comparisons of ROC plots were done with the Hanley & McNeil process. Power calculations were done post-hoc with Levene’s test. Analysis was done with R (R Core Team [2020]. R: A language and environment for statistical computing. R Foundation for Statistical Computing, Vienna, Austria. ISBN 3-900051-07-0, URL http://www.R-project.org/). Permission for this study was obtained from the Scientific Council/Ethics Board of the Evangelismos Hospital (No 170/April 24, 2020); we obtained informed consent for the inclusion of anonymized data from the patients or their next of kin.

## Results

**Figure 1:**
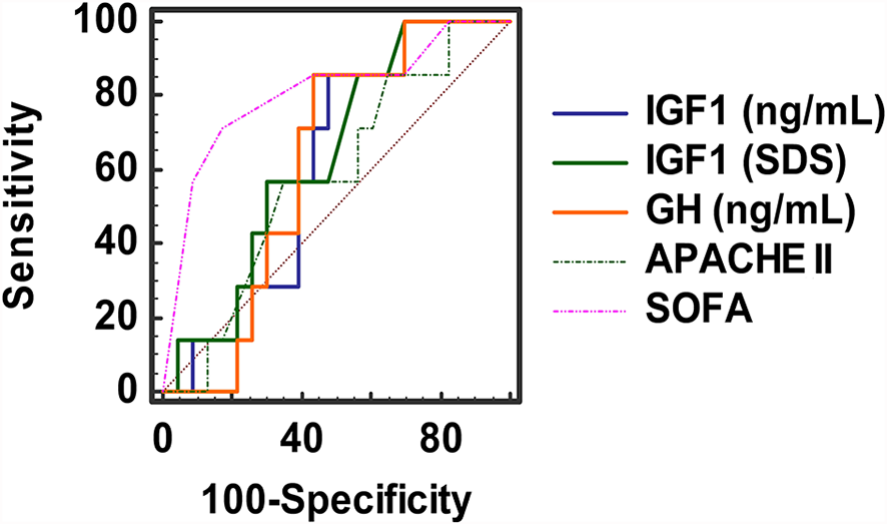
ROC plots of IGF-1 (raw data and SDS), GH, APACHE II and SOFA scores.

We studied 209 critically and non-critically ill patients with Covid-19; 39 wards’ patients with non-Covid-19 pneumonia served as contemporaneous controls. The patients’ demographics and laboratory evaluation results are presented in **Tables 1** and **2**. Patients who were ICU non-survivors were older and presented with a worse hematological/biochemical profile (including white blood cell count, troponin, glucose, aminotransferases and lactate dehydrogenase) compared to ICU survivors or Covid-19 survivors in the wards. Overall, no significant differences were noted in GH between all the groups, whereas IGF-1 SDS was higher in Covid-19 survivors compared to non-survivors (-0.96 ± 1.89 vs -2.05 ± 2.48, respectively, p=0.030); also the lowest IGF-1 SDS was in Covid-19 ICU non-survivors, whereas the highest was in non-Covid-19 controls that survived (**Table 1**). In critically and non-critically ill Covid-19 patients there were negative correlations between IGF1 and age (r= - 0.49, p<0.0001), C-reactive protein (CRP; r= - 0.34, p<0.0001), LDH (r=-0.38, p<0.0001), % of lymphocytes (r=-0.22, p=0.01) and % of neutrophils (r=-0.22, p=0.01). In a ROC plot of IGF-1/IGF-1 SDS or GH versus survival in the ICU patients, the areas under the curve (AUC) for IGF-1/IGF-1 SDS were similar to that of scores of well-established prognostic tools (namely the Acute Physiologic Assessment and Chronic Health Evaluation II; APACHE II, and Sequential Organ Failure Assessment; SOFA scores), whereas a marginal difference was noted for GH versus SOFA (**Figure 1** and **Table 3**). Regarding the analysis of IGF-1/IGF-1 SDS levels, as assessed in four (Covid-19 only) or six groups (Covid-19 and non-Covid-19 controls), the attained power was 0.662 or 0.697 and 0.658 or 0.694, respectively, whereas for GH the power was 0.546 or 0.475, respectively.

**Table 1:** Demographics, clinical and laboratory data of the study’s patients; data are presented as means (standard deviation), median (25^th^ percentile/75^th^ percentile) or counts.

Parameter	[1]. Covid-19-ICU-survivors (n=69)	[2]. Covid-19-ICU non-survivors (n=30)	[3]. Covid-19-Ward-survivors (n=104)	[4]. Covid-19-Ward non-survivors (n=6)	[5]. Non-Covid-cotrols-survivors (n=33)	[6]. Non-Covid-19-controls-non-survivors (n=6)
Gender (men/women)	52/17	24/6	71/33	3/3	23/10	4/2
Age (years)	62 (12)*	71 (10)	58 (18)*	58 (18)	66 (15)	73 (9)
Body Mass Index	27 (2)	26 (2)	26 (2)	26 (1)	27 (4)	25 (3)
Hypertension	23	15	30	4	10	1
More than one comorbidity#	19	14	28	4	20	4
APACHE II	15 (5)	17 (5)	N.A.	N.A.	N.A.	N.A.
SOFA	6 (4/8)	6 (4/9)	N.A.	N.A.	N.A.	N.A.
Growth Hormone (ng/mL)	0.33 (0.17/1.281)	0.69 (0.55/1.21)	0.22 (0.12/0.49)**	0.30 (0.13/1.22)	0.63 (0.31/1.17)	0.52 (0.41/0.83)
IGF 1 (ng/mL)	65.0 (41.7/103.2)	63.5 (32.0/88.0)	85.0( 63.0/133.8)	50.0 (35.9/119.3)	80.3 (67.8/108.5)	62.5 (50.9/89.6)
IGF 1 (SDS)	-1.39 (1.89)	-2.31 (1.92)	-0.84 (1.95)	-2.23 (2.93)	-0.15(1.42)+	-1.05(0.96)

APACHE II, Acute Physiologic Assessment and Chronic Health Evaluation II; SOFA, Sequential Organ Failure Assessment; #Other comorbidities: Smoking, Asthma, Chronic Obstructive Pulmonary Disease, Hyperlipidemia, Diabetes, Coronary Artery Disease, Chronic Kidney Disease, Hepatitis/Hepatic Failure; SDS, standard deviation score;

*[1,3] vs [2,4,5]; **[3] vs [2,4,5]; +[1,2,4] vs [5]; all p<0.05, Analysis of Variance or Kruskal-Wallis; N.A., Not Available.

**Table 2:** Clinical and laboratory data of the study’s Covid-19 patients; data are presented as means (standard deviation), median (25^th^ percentile/75^th^ percentile) or counts.

Parameter	[1]. Covid-19-ICU-survivors (n=69)	[2]. Covid-19-ICU non-survivors (n=30)	[3]. Covid-19-Ward-survivors (n=104)	[4]. Covid-19-Ward non-survivors (n=6)
Temperature	37.7 (37.3/38.1)	37.9 (37.5/38.5)	36.9 (37.6/38.0)	37.1 (36.8/37.2)
Mean Arterial BP (mmHg)	80 (11)	83 (16)	81 (9)	92 (12)
Heart Rate (bpm)	88 (17)	95 (22)	90 (12)	90 (4)
Respiratory Rate (per minute)	23 (20/26)	25 (22/27)	21 (19/25)*	25 (21/28)
White Blood Cell count (per μL)	8740 (6550/11150)	12300 (8480/17625)**	5810 (4280/7980)***	7830 (6102/9070)
Neutrophils%	84.0 (77.2/87.5)	88.4 (82.0/93.5)**	71.0 (61.5/82.6)***	76.9 (70.7/83.6)
Lymphocytes%	10.5 (7.0/15.0)	6.7 (3.7/11.5)**	22.0 (14.0/30.2)***	16.4 (10.2/21.6)
Hemoglobin (g/dL)	14.9 (14.8)	12.1 (1.8)	18.8 (26.6)	13.7 (1.1)
Platelets x1000 (per μL)	236 (177/293)	235 (199/313)	212 (162/269)	290 (237/299)
Fibrinogen (mg/dL)	619.8 (178.4)	664.8 (183.5)	518.5 (175.2)*	576.2 (147.1)
C-Reactive Protein (mg/L)	13.7 (14.7)	17.3 (10.5)	15.0 (27.2)	4.9 (4.5)
D Dimers (μg/mL)	1.72 (2.47)	1.57 (1.15)	7.00 (17.42)	0.52 (0.10)
Troponin (ng/mL)	12 (8/22)	48 (13/200)**	7 (5/16)***	8 (7/9)
Glucose (mg/dL)	141 (113/187)	152 (127/198)	103 (86/128)*	112 (90/132)
Creatinine (mg/dL)	0.90 (0.70/1.00)	1.10 (8.80/1.32)+	0.90 (0.70/1.00)	0.80 (0.72/0.87)
Aspartate Aminotransferase (U/L)	32 (42/60)++	34 (54/79)++	23 (30/52)	21 (33/36)
Alanine Aminotransferase (U/L)	41 (27/65)++	41 (26/68)++	27 (18/45)	19 (14/32)
Total Bilirubin (mg/dL)	0.57 (0.38/0.80)	0.55 (0.37/0.75)	0.43 (0.31/0.60)	0.55 (0.49/0.63)
Blood Urea Nitrogen (mg/dL)	37.0 (27.0/49.0)	54.0 (37.7/88.5)**	29.0 (24.0/36.0)***	29.0 (26.2/31.7)
Lactate Dehydrogenase (U/L)	414 (310/504)	514 (389/681)**	277 (217/850)***	257 (190/372)

*[3]vs[1,2]; **[2]vs[1,3,4]; ***[3]vs[1]; +[2]vs[1]; ++[1&2]vs[3&4]; all p<0.05, Analysis of Variance or Kruskal-Wallis.

**Table 3:** ROC plot analysis of prognostic tools’ scores and IGF1/GH versus survival in ICU patients with Covid-19.

	Area Under the Curve	Standard Error	95% Confidence Interval	Criterion value at maximum Youden’s J index (sensitivity; specificity; J index)	Criterion at 90% sensitivity
[1]. IGF1 (ng/mL)	0.698	0.112	0.480 to 0.917	88.0 (82%; 41%; 0.22)	99.0
[2]. IGF1 (SDS)	0.714	0.112	0.495 to 0.934	-3.10 (36%; 83%; 0.19)	-0.20
[3]. GH (ng/mL)	0.714	0.094	0.529 to 0.900	0.33 (86%; 56%; 0.42)	0.18
[4]. APACHE II (score)	0.611	0.115	0.385 to 0.837	15 (65%; 60%; 0.25)	11
[5]. SOFA (score)	0.889	0.067	0.757 to 0.999	2 (98%; 30%; 0.28)	3

[3] vs [5]: p = 0.061, [4] vs [5]: p = 0.014, Hanley & McNeil; SDS, standard deviation score.

## Discussion

In this observational study we noted lower IGF-1 SDS in critically ill Covid-19 patients compared to their counterparts with less severe disease or without Covid-19. Of importance, we also found that non-survivors had lower IGF-1 SDS than survivors.

GH and IGF1, the most important mediator of GH effects, apart their role in growth regulation during childhood, coordinate several processes throughout adult life (7, 8). GH stimulates protein synthesis, increases lipolysis, and potentiates the action of insulin (9). Moreover, IGF1 has been found to be pro-survival/anti-aging, anti-inflammatory, and antioxidant with neuro- and hepatoprotective properties (10). IGF-1 exerts beneficial effects over mitochondria by preserving it from the oxidative damage (10). Although the majority of data on the relation between GH and the immune system are from animal studies, it seems that GH may possess useful immunomodulatory actions. Normal aging is associated with a gradual decline in serum GH and the decline is more rapid in men than in women (10). Of note, the susceptibility of Covid-19 infection has been reported to be gender- and age-dependent and immune system dysfunction has been found to play a key role for poor clinical outcome (11–13). Despite these, the specific question about the impact of Covid-19 on GH/IGF1 axis remains currently unanswered. The only issue that has been previously addressed is whether the Covid-19 pandemic has affected the treatment adherence in patients with known GH deficiency (14). In our study, we measured GH and IGF-1 in a relatively large series with mild-to-critical Covid-19. We found that IGF-1 SDS levels were lower in Covid-19 patients who did not survive (particularly the ones who were mechanically ventilated). Moreover, IGF1 was inversely related with age, and markers of Covid-19 severity, i.e. LDH, CRP, % of lymphocytes and neutrophils. Sarcopenia in malnutrition/cachexia may be associated with lowering of IGF1 levels in muscle tissue or plasma (15, 16). Patients with Covid-19 are often malnourished (17) and with clinical sarcopenia. The latter is also attributed to excess cytokines’ production and action, disease severity status and immobilization (18). These causes (and in particular malnutrition) may also explain - in part - the IGF1 findings in our patients. Taken together, our results raise the possibility that there might be a link between low IGF-1, Covid-19 severity and poor outcome. To our knowledge, such findings have not been previously reported.

In critically ill patients, although GH secretion increases, hepatic GH receptors become downregulated and circulating IGF1 has been shown to decrease appreciably (5, 19). Prognostic value has been suggested for GH versus ICU survival in the past (20); in our patients, IGF1 vis-à-vis prognosis was on a par with accepted prognostic tools (Acute Physiologic Assessment and Chronic Health Evaluation II; APACHE II (21)and Sequential Organ Failure Assessment; SOFA (22)) and GH had a significantly higher AUC in ROC plot analysis compared to SOFA, a widely accepted scoring system assessing degree of organ dysfunction in ICU patients.

Older, prospective, randomized, placebo-controlled trials showed unexpectedly that the administration of recombinant human GH (rhGH) was associated with a marked increase in mortality in the rhGH group (23, 24). The main explanation for this untoward result was considered to be the high dosage of rhGH (24).

Our study lends credence to the known finding of the negative effect of age on IGF-1 (25), and the finding of lower mortality in patients with Covid-19 that have IGF-1 in the highest quartile compared to those patients that have IGF-1 in the lowest quartile (3) (though the latter finding was presented with no regard to severity of disease or localization of hospitalization i.e. wards or ICU). Of note, in a small (n=10) recent proteomic analysis study of plasma from critically ill Covid-19 patients, increased expression of the IGF-1 receptor was found, but no association was reported with regards to survival (4).

In the ever-shifting vista of Covid-19 many diagnostic and therapeutic means have been assessed in an effort to understand disease pathogenesis and tame the pandemic. Since studies of patients with Covid-19 and information on GH/IGF-1 (and their possible effect on disease outcomes) are scarce, we believe that this study contributes to the understanding of the disease. The study does not assess the presence of other associated disorders that may have contributed to the reduction of IGF-1 such as obesity, hepatic and renal dysfunction or acidosis. Any interference of medications such as anticoagulants in the measurement methods was not evaluated; IGF binding proteins or heterophilic antibodies (known to interfere with the measurement of IGF-1) were also not assessed. One could argue that the findings may be due only to the age of the patients, since IGF-1 is known to decrease with advanced age, although differences among the study groups were more pronounced with age/gender normalized IGF-1 SDS compared to raw IGF-1 levels. It is true that any cost-effectiveness or cost-benefit of measuring IGF-1/GH was not appraised in our work; we also have to acknowledge this as a limitation of this study. Another limitation of this study is that, at this sample size, the (a posteriori calculated) attained power was rather low.

In conclusion, our findings suggest that there might be an association between low IGF1 (and possibly GH) and poor outcome in patients with Covid-19. Thus, the question whether GH supplementation at appropriate dosages could be of therapeutic benefit in specific (possibly older) patients with Covid-19 and with concomitant GH deficiency, receiving ICU support, seems to be plausible.

## Data Availability Statement

The raw data supporting the conclusions of this article will be made available by the authors, without undue reservation.

## Ethics Statement

The studies involving human participants were reviewed and approved by the Scientific Council/Ethics Board of the Evangelismos Hospital (No 170/April 24, 2020). The patients/participants provided their written informed consent to participate in this study.

## Author Contributions

Conceptualization: II and ID. Methodology: II and ID. Software: II. Validation: II and ID. Formal analysis: II. Investigation: II, AD, EB, EJ, NA, STsi, AZ, AV, DV, AK and ID. Resources: AK, STsa and ID. Data curation: II, EJ and ID. Writing—original draft preparation: II and ID. Writing—review and editing: II and ID. Supervision: STsa and ID. Project administration: II, AK, and ID. All authors contributed to the article and approved the submitted version.

## Conflict of Interest

The authors declare that the research was conducted in the absence of any commercial or financial relationships that could be construed as a potential conflict of interest.
